# Tetra­kis(μ-4-*tert*-butyl­benzoato)-κ^4^ *O*:*O*′;κ^3^ *O*,*O*′:*O*′;κ^3^ *O*:*O*,*O*′-bis­[aqua­(4-*tert*-butyl­benzoato-κ^2^ *O*,*O*′)(4-*tert*-butyl­benzoic acid-κ*O*)neodymium(III)]

**DOI:** 10.1107/S1600536811015194

**Published:** 2011-05-07

**Authors:** Juan Yang, Jun Dai

**Affiliations:** a Department of Physics and Chemistry, Henan Polytechnic University, Jiaozuo 454003, People’s Republic of China; b Institute of Safety Science and Engineering, Henan Polytechnic University, Jiaozuo 454003, People’s Republic of China

## Abstract

The reaction of neodymium nitrate and 4-*tert*-butyl­benzoic acid (*t*BBAH) in aqueous solution yielded the dinuclear title complex, [Nd_2_(C_11_H_13_O_2_)_6_(C_11_H_14_O_2_)_2_(H_2_O)_2_], which has non-crystallographic *C_i_* symmetry. The two Nd^III^ ions are linked by two bridging and two bridging–chelating *t*BBA ligands with an Nd⋯Nd separation of 4.0624 (5) Å. Moreover, each Nd^III^ ion is coordinated by one chelating *t*BBA ion, one monodentate *t*BBAH ligand and one water mol­ecule. The nine-coordinated Nd^III^ ion is in a distorted tricapped trigonal–prismatic environment. The mol­ecules are linked into infinite chains along the *c* axis by inter­molecular O—H⋯O hydrogen bonds. Three of the *tert*-butyl groups are disordered over two sets of sites with equal occupancies.

## Related literature

For the structures and properties of lanthanide benzoate complexes, see: Roh *et al.* (2005 ▶); Singh *et al.* (2007 ▶); Xu *et al.* (2009 ▶); Yang *et al.* (2010 ▶). For geometrical parameters of compounds with similar nine-coordinate Nd^III^ atoms, see: Xiao *et al.* (2008 ▶); Wang *et al.* (2009 ▶).
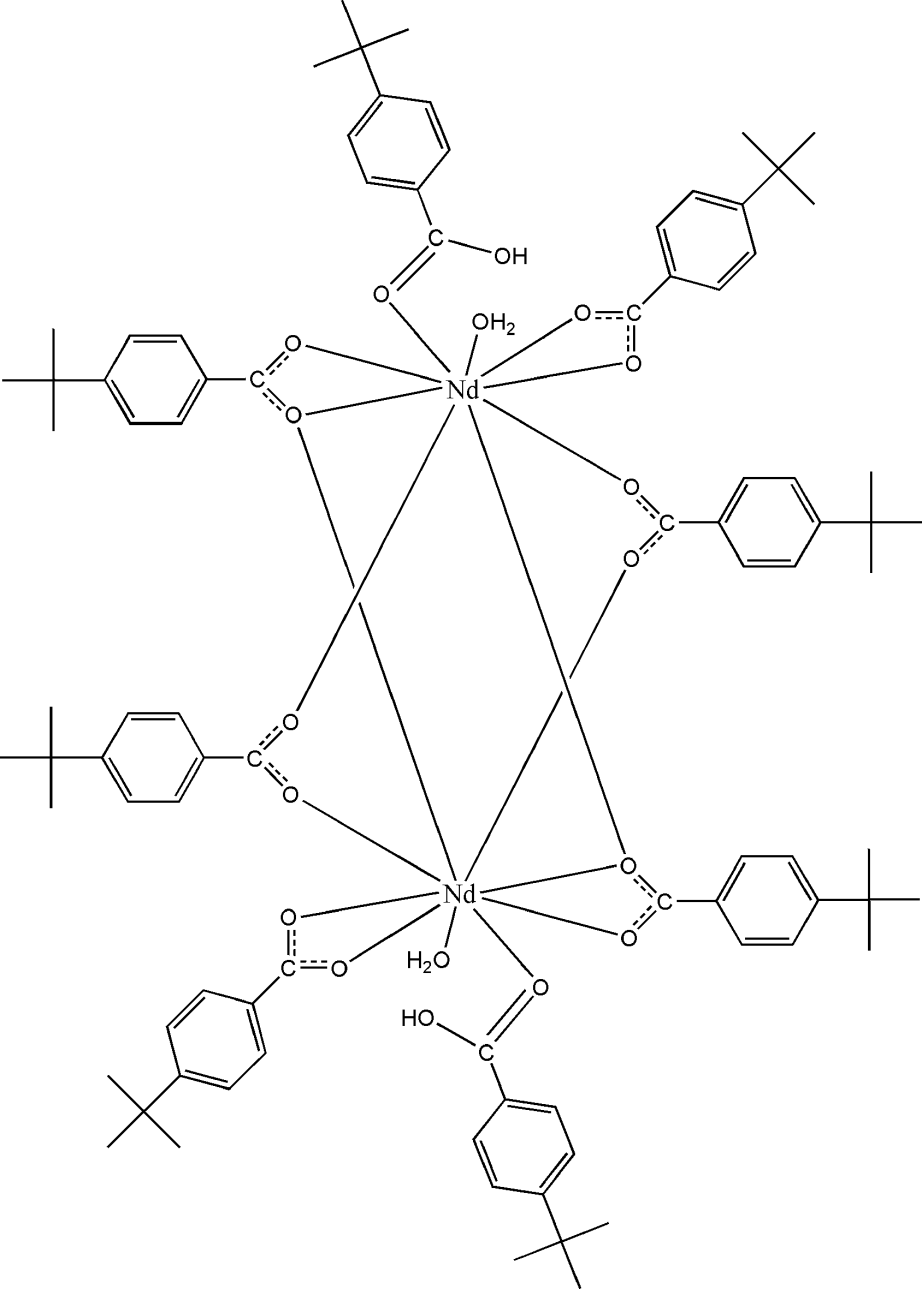


## Experimental

### 

#### Crystal data

[Nd_2_(C_11_H_13_O_2_)_6_(C_11_H_14_O_2_)_2_(H_2_O)_2_]
*M* *_r_* = 1744.24
Monoclinic, 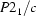

*a* = 35.261 (2) Å
*b* = 9.3563 (6) Å
*c* = 27.9406 (18) Å
β = 107.303 (1)°
*V* = 8800.8 (10) Å^3^
*Z* = 4
Mo *K*α radiation
μ = 1.23 mm^−1^
*T* = 296 K
0.25 × 0.15 × 0.05 mm

#### Data collection

Bruker APEXII CCD area-detector diffractometer
Absorption correction: multi-scan (*SADABS*; Bruker, 2007 ▶) *T*_min_ = 0.748, *T*_max_ = 0.941
48730 measured reflections
17410 independent reflections
12690 reflections with *I* > 2σ(*I*)
*R*_int_ = 0.061

#### Refinement

*R*[*F* ^2^ > 2σ(*F* ^2^)] = 0.061
*wR*(*F* ^2^) = 0.150
*S* = 1.06
17410 reflections
1091 parameters
1340 restraints
H atoms treated by a mixture of independent and constrained refinement
Δρ_max_ = 2.99 e Å^−3^
Δρ_min_ = −1.07 e Å^−3^

### 

Data collection: *APEX2* (Bruker, 2007 ▶); cell refinement: *SAINT* (Bruker, 2007 ▶); data reduction: *SAINT*; program(s) used to solve structure: *SHELXS97* (Sheldrick, 2008 ▶); program(s) used to refine structure: *SHELXL97* (Sheldrick, 2008 ▶); molecular graphics: *SHELXTL* (Sheldrick, 2008 ▶); software used to prepare material for publication: *SHELXTL*.

## Supplementary Material

Crystal structure: contains datablocks global, I. DOI: 10.1107/S1600536811015194/gk2367sup1.cif

Structure factors: contains datablocks I. DOI: 10.1107/S1600536811015194/gk2367Isup2.hkl

Additional supplementary materials:  crystallographic information; 3D view; checkCIF report

## Figures and Tables

**Table 1:** Selected bond lengths (Å)

Nd1—O7	2.376 (4)
Nd1—O5	2.397 (4)
Nd1—O1	2.405 (4)
Nd1—O3	2.445 (4)
Nd1—O17	2.518 (4)
Nd1—O9	2.524 (4)
Nd1—O11	2.539 (4)
Nd1—O10	2.578 (4)
Nd1—O4	2.804 (4)
Nd2—O6	2.356 (4)
Nd2—O8	2.357 (4)
Nd2—O4	2.426 (4)
Nd2—O2	2.471 (4)
Nd2—O14	2.530 (4)
Nd2—O18	2.536 (4)
Nd2—O15	2.544 (5)
Nd2—O13	2.572 (4)
Nd2—O1	2.761 (4)

**Table 2:** Hydrogen-bond geometry (Å, °)

*D*—H⋯*A*	*D*—H	H⋯*A*	*D*⋯*A*	*D*—H⋯*A*
O17—H17*A*⋯O13^i^	0.85	2.12	2.870 (6)	148
O17—H17⋯O2^i^	0.85	1.96	2.786 (6)	165
O18—H18*A*⋯O3^ii^	0.85	2.01	2.782 (6)	151
O18—H18*B*⋯O10^ii^	0.85	2.14	2.843 (6)	140

Symmetry codes: (i) 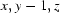; (ii) 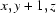.

**Fig. 1.**
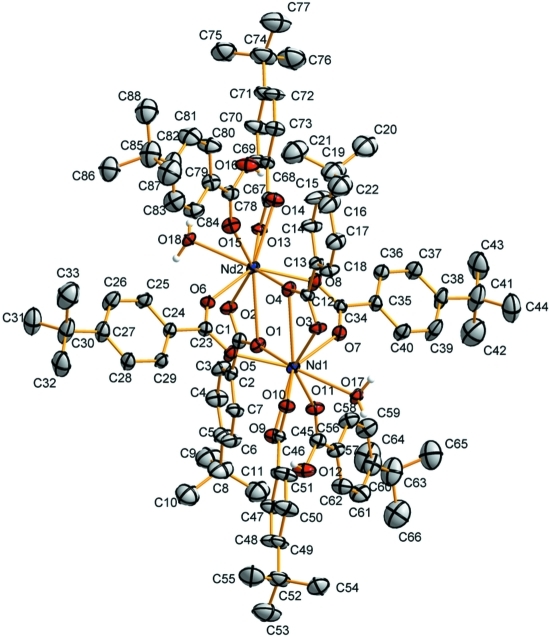
The molecular structure of the title complex. Displacement ellipsoids for non-hydrogen atoms are drawn at the 30% probability level. All aryl and methyl type H-atoms have been omitted for clarity. Disorder of the tert-buthyl groups is not shown.

**Fig. 2.**
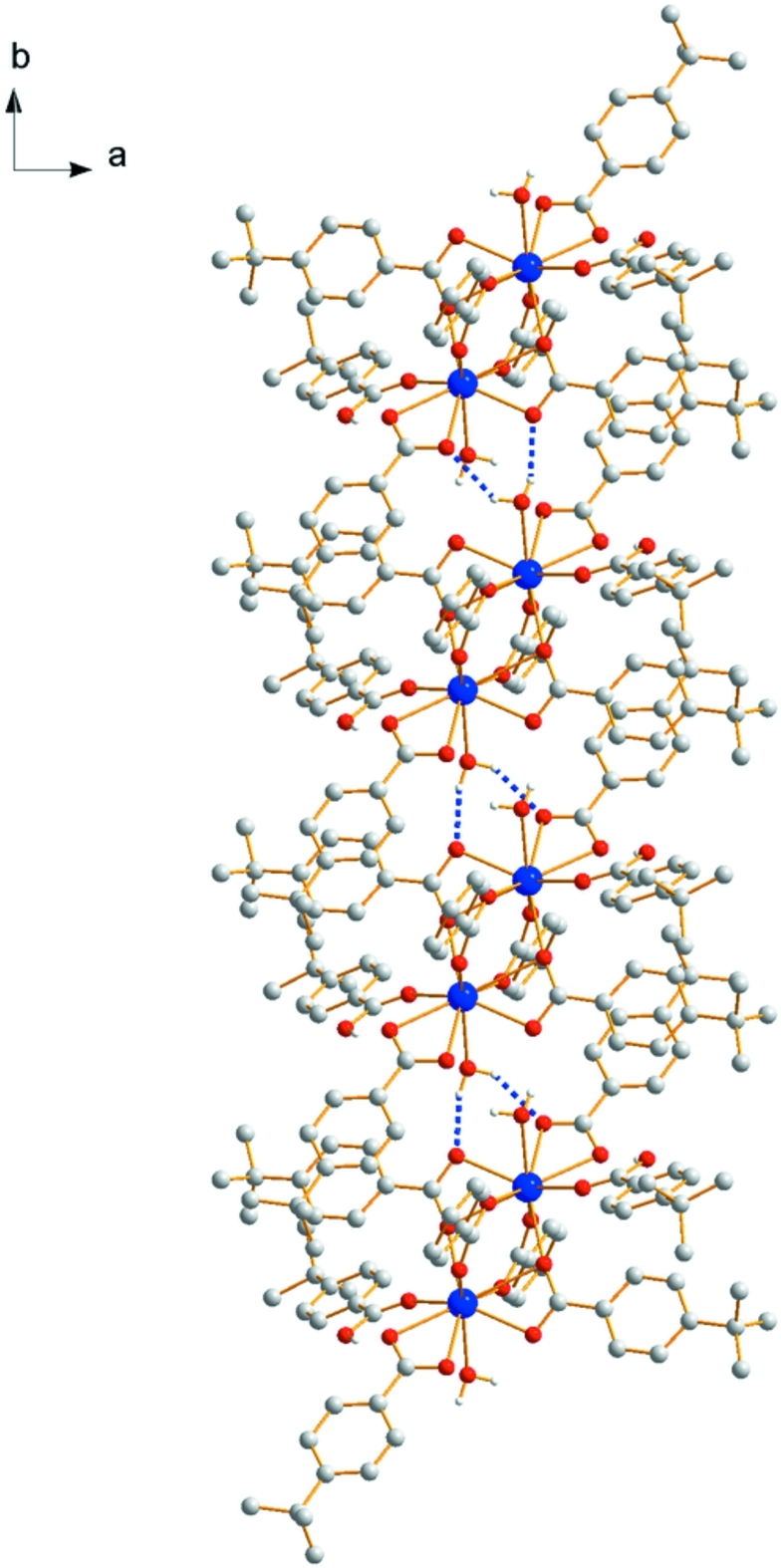
The hydrogen-bonded chain structure of the title compound, viewed along the c axis. Intermolecular hydrogen bonds are shown as dashed lines in blue.

## supplementary crystallographic information

### Comment

Lanthanide carboxylate complexes show interesting crystal structures due to the variable coordination number of metal centers as well as coordination versatility of carboxylate ligands. As rigid ligands, benzoic acid and its derivatives have been widely used for lanthanide complexes because they improve the thermal stability and luminescence (Roh *et al.*, 2005; Singh *et al.*, 2007; Xu *et al.*, 2009). In continuation to our research (Yang *et al.*, 2010), we now report the preparation and crystal structure of a new Nd^III^ complex obtained in the reaction with 4-*tert*-butylbenzoic acid (*t*BBAH).

The asymmetric unit of the title dinuclear complex (Fig.1) contains two Nd atoms, six *t*BBA ligands, two *t*BBAH ligands and two coordinated water molecules. The title molecule [Nd_2_(C_11_H_13_O_2_)_6_(C_11_H_14_O_2_)_2_(H_2_O)_2_] has a non-crystallographic *C_i_* symmetry. The *t*BBA ligands show four coordination modes: bridging bidentate, chelating bidentate, bridging-chelating tridentate and monodentate mode (*t*BBAH). The two Nd^III^ ions are linked by four bridging *t*BBA ligands, with a Nd···Nd seperation of 4.0624 (5) Å. Each Nd^III^ ion is additionally coordinated by one *O*,*O*'-bidentate *t*BBA ion, one monodentate *t*BBAH ligand and one water molecule. The Nd—O bond lengths are in the range 2.356 (4)  - 2.804 (4) Å, which is comparable to those reported for other Nd complexes with oxygen environment around the central metal (Xiao *et al.*, 2008; Wang *et al.*, 2009). Each nine-coordinated Nd^III^ ion adopts a distorted tricapped trigonal-prismatic geometry. The crystal packing is stabilized by intermolecular O—H···O hydrogen bonds (Table 2), which link the molecules into one-dimensional chains along the *c* axis (Fig.2).

### Experimental

A mixture of Nd(NO_3_)_3_^.^6H_2_O (0.228 g, 0.52 mmol), *t*BBAH (0.150 g, 0.84mmol), melamine (0.026 g, 0.20 mmol) and distilled water (10 ml) was sealed in a 25 ml Teflon-lined stainless autoclave. The mixture was heated at 423 K for 5 days to give the purple prism crystals suitable for X-ray diffraction analysis.

### Refinement

All H atoms bound to C atoms were placed in calculated positions and treated in a riding-model approxination, with C—H = 0.93 Å and 0.96 Å for aryl and methyl type H-atoms, respectively with *U*_iso_(H) = 1.2Ueq(C) or 1.5 *U*_eq_(C_methyl_). The H atoms of water molecules were located from difference maps and treated as riding with an idealized distance of O—H = 0.85 Å and *U*_iso_(H) = 1.5Ueq(O). The position of carboxylic H atoms were located in the difference Fourier maps and were included in the subsequent refinement using the restraint (O—H = 0.82 (2) Å and *U*_iso_(H) = 1.5Ueq(O)). Among 1340 restraints used in the refinement are mainly those used to restrain geometry of *tert*-butyl groups. Large thermal motion of the *tert*-butyl groups required that restraints to be applied to the thermal parameters, namely, SIMU 0.01 0.02 3.8 C8 > C11B; SIMU 0.01 0.02 3.8 C16 C19 > C22 B; SIMU 0.01 0.02 3.8 C30 > C33; SIMU 0.01 0.02 3.8 C41 > C44; SIMU 0.01 0.02 3.8 C52 > C55; SIMU 0.005 0.01 3.8 C63 > C66; SIMU 0.01 0.02 3.8 C74 > C77; SIMU 0.01 0.02 3.8 C85 > C88B. SHELXL-97 ISOR restraints are imposed on the displacement ellipsoids of the methyl groups, including C9 > C11B; C20 > C22B; C31 > C33; C42 > C44; C53 > C55; C64 > C66; C75 > C77; C86 > C88B. Some rigid-bond restraints to Uij (DELU) were imposed on bonded atoms, including C5-C8; C16-C19; C19-C20; C30-C33; C41-C42; C41-C43; C63-C64; C63-C65; C74-C77.

The highest residual peak of 2.99 e Å^-3^ is located at the distance of 1.18 Å from O13; the deepest hole -1.07 e Å^-3^ is at 0.89 Å from Nd1. The methyl groups of three *tert*-butyl groups [(H_3_C9,H_3_C10,H_3_C11),(H_3_C20,H_3_C21,H_3_C22),(H_3_C86,H_3_C87,H_3_C88)] are disordered over two sites with equal occupancies.

### Crystal data

**Table:**

[Nd_2_(C_11_H_13_O_2_)_6_(C_11_H_14_O_2_)_2_(H_2_O)_2_]	*F*(000) = 3608
*M**_r_* = 1744.24	*D*_x_ = 1.316 Mg m^−^^3^
Monoclinic, *P*2_1_/*c*	Mo *K*α radiation, λ = 0.71073 Å
Hall symbol: -P 2ybc	Cell parameters from 7572 reflections
*a* = 35.261 (2) Å	θ = 2.3–22.7°
*b* = 9.3563 (6) Å	µ = 1.23 mm^−^^1^
*c* = 27.9406 (18) Å	*T* = 296 K
β = 107.303 (1)°	Prism, purple
*V* = 8800.8 (10) Å^3^	0.25 × 0.15 × 0.05 mm
*Z* = 4	

### Data collection

**Table:**

Bruker APEXII CCD area-detector diffractometer	17410 independent reflections
Radiation source: fine-focus sealed tube	12690 reflections with *I* > 2σ(*I*)
graphite	*R*_int_ = 0.061
phi and ω scans	θ_max_ = 26.1°, θ_min_ = 1.5°
Absorption correction: multi-scan (*SADABS*; Bruker, 2007)	*h* = −43→43
*T*_min_ = 0.748, *T*_max_ = 0.941	*k* = −11→7
48730 measured reflections	*l* = −34→33

### Refinement

**Table:**

Refinement on *F*^2^	Primary atom site location: structure-invariant direct methods
Least-squares matrix: full	Secondary atom site location: difference Fourier map
*R*[*F*^2^ > 2σ(*F*^2^)] = 0.061	Hydrogen site location: inferred from neighbouring sites
*wR*(*F*^2^) = 0.150	H atoms treated by a mixture of independent and constrained refinement
*S* = 1.06	*w* = 1/[σ^2^(*F*_o_^2^) + (0.055*P*)^2^ + 26.*P*] where *P* = (*F*_o_^2^ + 2*F*_c_^2^)/3
17410 reflections	(Δ/σ)_max_ = 0.001
1091 parameters	Δρ_max_ = 2.99 e Å^−^^3^
1340 restraints	Δρ_min_ = −1.06 e Å^−^^3^

### Fractional atomic coordinates and isotropic or equivalent isotropic displacement parameters (Å^2^)

**Table:**

	*x*	*y*	*z*	*U*_iso_*/*U*_eq_	Occ. (<1)
Nd1	0.219995 (10)	0.29547 (3)	0.367934 (12)	0.02734 (10)	
Nd2	0.279016 (10)	0.67394 (3)	0.394574 (12)	0.02793 (10)	
O1	0.20676 (13)	0.5449 (4)	0.34956 (16)	0.0365 (10)	
O2	0.21436 (13)	0.7758 (4)	0.34609 (17)	0.0389 (11)	
O3	0.28392 (13)	0.1930 (4)	0.41530 (17)	0.0388 (11)	
O4	0.29378 (13)	0.4234 (4)	0.41380 (17)	0.0385 (11)	
O5	0.21695 (14)	0.4040 (5)	0.44441 (16)	0.0430 (12)	
O6	0.24304 (13)	0.6229 (5)	0.45133 (16)	0.0398 (11)	
O7	0.25571 (14)	0.3457 (5)	0.30995 (16)	0.0398 (11)	
O8	0.28176 (15)	0.5667 (5)	0.31933 (17)	0.0481 (12)	
O9	0.15420 (13)	0.1858 (5)	0.36625 (17)	0.0434 (12)	
O10	0.20547 (13)	0.0869 (5)	0.42019 (16)	0.0369 (10)	
O11	0.17127 (14)	0.3027 (5)	0.28033 (17)	0.0445 (12)	
O12	0.11414 (17)	0.1913 (7)	0.2726 (2)	0.0635 (16)	
H12	0.123 (3)	0.177 (11)	0.3027 (11)	0.095*	
O13	0.29331 (12)	0.8822 (5)	0.34240 (16)	0.0371 (10)	
O14	0.34480 (13)	0.7799 (5)	0.39364 (17)	0.0437 (11)	
O15	0.32861 (15)	0.6716 (5)	0.48188 (17)	0.0475 (12)	
O16	0.38704 (17)	0.7661 (7)	0.4863 (2)	0.0660 (16)	
H16	0.379 (3)	0.763 (12)	0.4558 (8)	0.099*	
O17	0.22459 (13)	0.0619 (4)	0.32520 (15)	0.0378 (10)	
H17A	0.2489	0.0417	0.3294	0.057*	
H17	0.2171	−0.0233	0.3279	0.057*	
O18	0.27352 (13)	0.9085 (4)	0.43754 (16)	0.0378 (10)	
H18A	0.2818	0.9809	0.4253	0.057*	
H18B	0.2487	0.9195	0.4322	0.057*	
C1	0.19241 (19)	0.6669 (6)	0.3350 (2)	0.0320 (14)	
C2	0.15077 (19)	0.6812 (7)	0.3044 (2)	0.0350 (15)	
C3	0.1377 (2)	0.8033 (8)	0.2768 (3)	0.0497 (19)	
H3A	0.1548	0.8808	0.2802	0.060*	
C4	0.1001 (2)	0.8130 (9)	0.2445 (3)	0.064 (2)	
H4A	0.0923	0.8966	0.2263	0.076*	
C5	0.0734 (2)	0.6998 (10)	0.2384 (4)	0.066 (2)	
C6	0.0862 (2)	0.5805 (9)	0.2684 (4)	0.067 (3)	
H6A	0.0687	0.5047	0.2666	0.080*	
C7	0.1237 (2)	0.5720 (8)	0.3006 (3)	0.0472 (18)	
H7A	0.1312	0.4908	0.3204	0.057*	
C8	0.0321 (3)	0.7079 (12)	0.1994 (4)	0.101 (3)	
C9	0.0231 (8)	0.849 (2)	0.1732 (10)	0.114 (5)	0.50
H9A	−0.0051	0.8608	0.1596	0.171*	0.50
H9B	0.0351	0.8531	0.1465	0.171*	0.50
H9C	0.0337	0.9250	0.1966	0.171*	0.50
C10	0.0013 (6)	0.699 (3)	0.2289 (8)	0.107 (5)	0.50
H10A	−0.0247	0.7195	0.2067	0.161*	0.50
H10B	0.0079	0.7679	0.2556	0.161*	0.50
H10C	0.0016	0.6050	0.2426	0.161*	0.50
C11	0.0267 (7)	0.582 (2)	0.1635 (8)	0.104 (5)	0.50
H11A	0.0001	0.5822	0.1410	0.156*	0.50
H11B	0.0312	0.4940	0.1822	0.156*	0.50
H11C	0.0453	0.5890	0.1445	0.156*	0.50
C9B	0.0089 (7)	0.826 (2)	0.2140 (10)	0.110 (5)	0.50
H9BA	−0.0165	0.8342	0.1892	0.164*	0.50
H9BB	0.0231	0.9139	0.2164	0.164*	0.50
H9BC	0.0051	0.8037	0.2459	0.164*	0.50
C10B	0.0120 (7)	0.564 (2)	0.1906 (10)	0.112 (5)	0.50
H10D	0.0086	0.5298	0.2214	0.168*	0.50
H10E	0.0280	0.4983	0.1789	0.168*	0.50
H10F	−0.0135	0.5735	0.1660	0.168*	0.50
C11B	0.0364 (7)	0.761 (3)	0.1490 (7)	0.108 (5)	0.50
H11D	0.0107	0.7658	0.1246	0.163*	0.50
H11E	0.0529	0.6956	0.1375	0.163*	0.50
H11F	0.0484	0.8540	0.1533	0.163*	0.50
C12	0.30664 (18)	0.2991 (7)	0.4274 (2)	0.0328 (14)	
C13	0.34812 (19)	0.2773 (7)	0.4587 (2)	0.0380 (15)	
C14	0.3759 (2)	0.3856 (8)	0.4652 (4)	0.067 (3)	
H14A	0.3692	0.4717	0.4482	0.080*	
C15	0.4132 (3)	0.3653 (10)	0.4967 (5)	0.107 (5)	
H15A	0.4313	0.4401	0.5011	0.128*	
C16	0.4257 (3)	0.2382 (13)	0.5227 (5)	0.103 (3)	
C17	0.3978 (2)	0.1298 (9)	0.5131 (3)	0.064 (2)	
H17B	0.4048	0.0414	0.5283	0.077*	
C18	0.3602 (2)	0.1483 (8)	0.4819 (3)	0.0487 (19)	
H18C	0.3424	0.0724	0.4763	0.058*	
C19	0.4673 (3)	0.2190 (13)	0.5612 (5)	0.112 (3)	
C20	0.4962 (7)	0.237 (3)	0.5308 (10)	0.121 (4)	0.50
H20A	0.4920	0.1622	0.5062	0.181*	0.50
H20B	0.5229	0.2313	0.5527	0.181*	0.50
H20C	0.4921	0.3280	0.5144	0.181*	0.50
C21	0.4707 (9)	0.336 (3)	0.6005 (9)	0.124 (5)	0.50
H21A	0.4657	0.4276	0.5842	0.186*	0.50
H21B	0.4970	0.3354	0.6237	0.186*	0.50
H21C	0.4517	0.3192	0.6182	0.186*	0.50
C22	0.4723 (7)	0.080 (2)	0.5918 (9)	0.110 (5)	0.50
H22A	0.4661	0.0001	0.5694	0.164*	0.50
H22B	0.4546	0.0816	0.6121	0.164*	0.50
H22C	0.4992	0.0728	0.6129	0.164*	0.50
C20B	0.4822 (7)	0.071 (2)	0.5552 (11)	0.125 (5)	0.50
H20D	0.5098	0.0641	0.5739	0.188*	0.50
H20E	0.4790	0.0530	0.5204	0.188*	0.50
H20F	0.4673	0.0019	0.5676	0.188*	0.50
C21B	0.4660 (9)	0.265 (3)	0.6124 (7)	0.123 (5)	0.50
H21D	0.4924	0.2651	0.6353	0.184*	0.50
H21E	0.4497	0.1998	0.6242	0.184*	0.50
H21F	0.4550	0.3595	0.6104	0.184*	0.50
C22B	0.4977 (7)	0.312 (3)	0.5457 (10)	0.119 (5)	0.50
H22D	0.5241	0.2800	0.5630	0.178*	0.50
H22E	0.4947	0.4099	0.5542	0.178*	0.50
H22F	0.4932	0.3039	0.5102	0.178*	0.50
C23	0.22736 (19)	0.5196 (7)	0.4677 (2)	0.0364 (15)	
C24	0.22088 (19)	0.5389 (7)	0.5173 (2)	0.0342 (14)	
C25	0.2371 (2)	0.6567 (8)	0.5466 (3)	0.0478 (18)	
H25A	0.2516	0.7231	0.5345	0.057*	
C26	0.2320 (3)	0.6767 (8)	0.5926 (3)	0.062 (2)	
H26A	0.2437	0.7550	0.6118	0.074*	
C27	0.2094 (3)	0.5809 (8)	0.6113 (3)	0.056 (2)	
C28	0.1930 (2)	0.4656 (8)	0.5818 (3)	0.052 (2)	
H28A	0.1776	0.4008	0.5931	0.063*	
C29	0.1989 (2)	0.4442 (8)	0.5357 (3)	0.0444 (17)	
H29A	0.1879	0.3644	0.5168	0.053*	
C30	0.2046 (4)	0.6064 (9)	0.6624 (4)	0.084 (2)	
C31	0.1917 (4)	0.7553 (11)	0.6684 (4)	0.104 (3)	
H31A	0.1648	0.7684	0.6480	0.155*	
H31B	0.2086	0.8215	0.6582	0.155*	
H31C	0.1935	0.7719	0.7029	0.155*	
C32	0.1828 (3)	0.4913 (11)	0.6802 (4)	0.096 (3)	
H32A	0.1562	0.4846	0.6581	0.144*	
H32B	0.1821	0.5134	0.7134	0.144*	
H32C	0.1962	0.4018	0.6806	0.144*	
C33	0.2478 (4)	0.5994 (14)	0.7035 (4)	0.121 (4)	
H33A	0.2452	0.6187	0.7362	0.182*	
H33B	0.2649	0.6695	0.6957	0.182*	
H33C	0.2589	0.5059	0.7033	0.182*	
C34	0.27166 (18)	0.4509 (7)	0.2955 (2)	0.0324 (14)	
C35	0.2789 (2)	0.4393 (7)	0.2451 (2)	0.0360 (15)	
C36	0.3002 (2)	0.5411 (7)	0.2290 (3)	0.0425 (17)	
H36A	0.3104	0.6193	0.2492	0.051*	
C37	0.3064 (3)	0.5278 (8)	0.1827 (3)	0.057 (2)	
H37A	0.3208	0.5980	0.1722	0.068*	
C38	0.2919 (3)	0.4138 (8)	0.1517 (3)	0.060 (2)	
C39	0.2692 (3)	0.3130 (9)	0.1682 (3)	0.073 (3)	
H39A	0.2583	0.2359	0.1478	0.088*	
C40	0.2631 (3)	0.3263 (8)	0.2138 (3)	0.055 (2)	
H40A	0.2479	0.2582	0.2240	0.066*	
C41	0.2968 (4)	0.3975 (10)	0.0995 (4)	0.098 (3)	
C42	0.2539 (4)	0.4092 (16)	0.0600 (5)	0.138 (4)	
H42A	0.2558	0.3937	0.0268	0.206*	
H42B	0.2432	0.5027	0.0619	0.206*	
H42C	0.2367	0.3384	0.0674	0.206*	
C43	0.3168 (4)	0.5183 (12)	0.0838 (4)	0.116 (3)	
H43A	0.3438	0.5235	0.1048	0.174*	
H43B	0.3032	0.6054	0.0867	0.174*	
H43C	0.3164	0.5051	0.0496	0.174*	
C44	0.3090 (4)	0.2504 (12)	0.0905 (4)	0.116 (3)	
H44A	0.3353	0.2319	0.1122	0.174*	
H44B	0.3089	0.2416	0.0562	0.174*	
H44C	0.2908	0.1827	0.0971	0.174*	
C45	0.16890 (18)	0.0877 (7)	0.3971 (2)	0.0320 (14)	
C46	0.14351 (19)	−0.0307 (7)	0.4057 (2)	0.0358 (15)	
C47	0.1039 (2)	−0.0367 (9)	0.3802 (3)	0.055 (2)	
H47A	0.0923	0.0361	0.3580	0.067*	
C48	0.0806 (2)	−0.1505 (9)	0.3872 (3)	0.063 (2)	
H48A	0.0536	−0.1516	0.3696	0.076*	
C49	0.0961 (2)	−0.2604 (8)	0.4190 (4)	0.062 (2)	
C50	0.1356 (3)	−0.2533 (9)	0.4435 (4)	0.073 (3)	
H50A	0.1471	−0.3270	0.4653	0.088*	
C51	0.1592 (2)	−0.1429 (8)	0.4377 (3)	0.057 (2)	
H51A	0.1861	−0.1433	0.4553	0.069*	
C52	0.0716 (3)	−0.3871 (10)	0.4287 (4)	0.086 (3)	
C53	0.0281 (3)	−0.3664 (13)	0.4013 (5)	0.123 (4)	
H53A	0.0245	−0.3606	0.3659	0.184*	
H53B	0.0189	−0.2796	0.4124	0.184*	
H53C	0.0132	−0.4458	0.4080	0.184*	
C54	0.0860 (3)	−0.5240 (10)	0.4109 (4)	0.090 (3)	
H54A	0.0841	−0.5153	0.3760	0.135*	
H54B	0.0699	−0.6025	0.4155	0.135*	
H54C	0.1132	−0.5408	0.4300	0.135*	
C55	0.0762 (4)	−0.4029 (13)	0.4842 (4)	0.115 (4)	
H55A	0.0581	−0.4743	0.4889	0.173*	
H55B	0.0705	−0.3132	0.4973	0.173*	
H55C	0.1029	−0.4310	0.5016	0.173*	
C56	0.1389 (2)	0.2629 (7)	0.2543 (3)	0.0427 (16)	
C57	0.1242 (2)	0.2930 (7)	0.2004 (3)	0.0428 (17)	
C58	0.1474 (3)	0.3752 (9)	0.1789 (3)	0.061 (2)	
H58A	0.1715	0.4113	0.1987	0.074*	
C59	0.1348 (3)	0.4038 (10)	0.1284 (3)	0.071 (3)	
H59A	0.1507	0.4599	0.1147	0.086*	
C60	0.0997 (3)	0.3523 (9)	0.0975 (3)	0.061 (2)	
C61	0.0774 (3)	0.2691 (11)	0.1193 (3)	0.069 (3)	
H61A	0.0534	0.2318	0.0994	0.082*	
C62	0.0895 (2)	0.2394 (10)	0.1699 (3)	0.061 (2)	
H62A	0.0737	0.1819	0.1834	0.073*	
C63	0.0867 (4)	0.3878 (12)	0.0421 (4)	0.107 (3)	
C64	0.0784 (4)	0.5455 (13)	0.0341 (5)	0.133 (3)	
H64A	0.0688	0.5651	−0.0012	0.200*	
H64B	0.1025	0.5983	0.0486	0.200*	
H64C	0.0588	0.5735	0.0498	0.200*	
C65	0.1164 (4)	0.3366 (15)	0.0172 (4)	0.123 (3)	
H65A	0.1061	0.3511	−0.0184	0.184*	
H65B	0.1215	0.2367	0.0240	0.184*	
H65C	0.1407	0.3893	0.0299	0.184*	
C66	0.0478 (4)	0.3144 (15)	0.0139 (4)	0.129 (3)	
H66A	0.0410	0.3386	−0.0211	0.194*	
H66B	0.0271	0.3458	0.0272	0.194*	
H66C	0.0509	0.2128	0.0177	0.194*	
C67	0.3293 (2)	0.8817 (7)	0.3634 (2)	0.0321 (14)	
C68	0.35482 (19)	1.0016 (7)	0.3572 (2)	0.0356 (15)	
C69	0.3384 (2)	1.1149 (8)	0.3259 (3)	0.057 (2)	
H69A	0.3115	1.1135	0.3082	0.068*	
C70	0.3613 (2)	1.2295 (9)	0.3206 (4)	0.077 (3)	
H70A	0.3494	1.3048	0.2998	0.092*	
C71	0.4012 (3)	1.2360 (9)	0.3451 (4)	0.071 (3)	
C72	0.4180 (2)	1.1219 (10)	0.3759 (3)	0.066 (2)	
H72A	0.4451	1.1222	0.3928	0.079*	
C73	0.3947 (2)	1.0077 (9)	0.3817 (3)	0.053 (2)	
H73A	0.4065	0.9330	0.4028	0.064*	
C74	0.4259 (3)	1.3644 (11)	0.3373 (5)	0.109 (3)	
C75	0.4083 (3)	1.5008 (11)	0.3521 (5)	0.108 (3)	
H75A	0.4102	1.4968	0.3871	0.161*	
H75B	0.3810	1.5091	0.3326	0.161*	
H75C	0.4229	1.5821	0.3461	0.161*	
C76	0.4225 (4)	1.3779 (15)	0.2818 (5)	0.137 (4)	
H76A	0.4336	1.2946	0.2711	0.205*	
H76B	0.4366	1.4613	0.2766	0.205*	
H76C	0.3950	1.3862	0.2628	0.205*	
C77	0.4692 (3)	1.3478 (14)	0.3632 (6)	0.138 (4)	
H77A	0.4738	1.3490	0.3989	0.207*	
H77B	0.4835	1.4252	0.3539	0.207*	
H77C	0.4783	1.2587	0.3536	0.207*	
C78	0.3618 (2)	0.7065 (8)	0.5066 (3)	0.0463 (17)	
C79	0.3772 (2)	0.6833 (8)	0.5610 (3)	0.0483 (18)	
C80	0.4140 (3)	0.7277 (11)	0.5882 (3)	0.073 (3)	
H80A	0.4306	0.7716	0.5723	0.087*	
C81	0.4268 (3)	0.7077 (12)	0.6397 (4)	0.084 (3)	
H81A	0.4520	0.7386	0.6580	0.101*	
C82	0.4028 (3)	0.6427 (11)	0.6642 (3)	0.074 (3)	
C83	0.3659 (3)	0.5984 (11)	0.6360 (3)	0.077 (3)	
H83A	0.3492	0.5550	0.6519	0.093*	
C84	0.3530 (3)	0.6167 (9)	0.5846 (3)	0.063 (2)	
H84A	0.3281	0.5840	0.5661	0.075*	
C85	0.4173 (3)	0.6198 (13)	0.7221 (4)	0.098 (3)	
C86	0.3872 (6)	0.687 (3)	0.7456 (8)	0.100 (5)	0.50
H86D	0.3960	0.6723	0.7812	0.150*	0.50
H86E	0.3617	0.6432	0.7316	0.150*	0.50
H86F	0.3852	0.7878	0.7386	0.150*	0.50
C87	0.4206 (9)	0.4614 (18)	0.7344 (9)	0.116 (5)	0.50
H87D	0.4335	0.4488	0.7696	0.173*	0.50
H87E	0.4358	0.4150	0.7156	0.173*	0.50
H87F	0.3944	0.4203	0.7259	0.173*	0.50
C88	0.4567 (6)	0.694 (3)	0.7476 (9)	0.111 (5)	0.50
H88D	0.4610	0.6967	0.7832	0.166*	0.50
H88E	0.4559	0.7904	0.7352	0.166*	0.50
H88F	0.4780	0.6432	0.7405	0.166*	0.50
C86B	0.3850 (6)	0.559 (3)	0.7410 (9)	0.115 (5)	0.50
H86A	0.3950	0.5440	0.7766	0.173*	0.50
H86B	0.3763	0.4688	0.7248	0.173*	0.50
H86C	0.3630	0.6237	0.7338	0.173*	0.50
C87B	0.4528 (6)	0.516 (3)	0.7333 (8)	0.105 (5)	0.50
H87A	0.4627	0.4995	0.7688	0.158*	0.50
H87B	0.4735	0.5561	0.7217	0.158*	0.50
H87C	0.4443	0.4266	0.7165	0.158*	0.50
C88B	0.4319 (8)	0.761 (2)	0.7470 (9)	0.119 (5)	0.50
H88A	0.4381	0.7510	0.7827	0.179*	0.50
H88B	0.4116	0.8323	0.7355	0.179*	0.50
H88C	0.4553	0.7901	0.7387	0.179*	0.50

### Atomic displacement parameters (Å^2^)

**Table:**

	*U*^11^	*U*^22^	*U*^33^	*U*^12^	*U*^13^	*U*^23^
Nd1	0.03261 (19)	0.01953 (16)	0.02934 (18)	−0.00264 (13)	0.00839 (14)	0.00240 (13)
Nd2	0.03464 (19)	0.01989 (16)	0.02951 (18)	−0.00302 (13)	0.00993 (14)	0.00167 (13)
O1	0.041 (3)	0.026 (2)	0.037 (2)	0.003 (2)	0.004 (2)	0.005 (2)
O2	0.038 (3)	0.023 (2)	0.050 (3)	−0.001 (2)	0.005 (2)	0.006 (2)
O3	0.036 (2)	0.025 (2)	0.050 (3)	−0.005 (2)	0.005 (2)	0.005 (2)
O4	0.044 (3)	0.024 (2)	0.047 (3)	0.003 (2)	0.013 (2)	0.003 (2)
O5	0.064 (3)	0.031 (3)	0.039 (3)	−0.011 (2)	0.025 (2)	−0.005 (2)
O6	0.048 (3)	0.034 (2)	0.043 (3)	−0.008 (2)	0.022 (2)	−0.001 (2)
O7	0.056 (3)	0.029 (2)	0.041 (3)	−0.005 (2)	0.025 (2)	0.002 (2)
O8	0.074 (4)	0.035 (3)	0.043 (3)	−0.012 (2)	0.030 (3)	−0.006 (2)
O9	0.042 (3)	0.043 (3)	0.043 (3)	−0.004 (2)	0.009 (2)	0.014 (2)
O10	0.040 (3)	0.031 (2)	0.040 (3)	−0.004 (2)	0.013 (2)	0.009 (2)
O11	0.041 (3)	0.043 (3)	0.042 (3)	−0.005 (2)	0.002 (2)	0.007 (2)
O12	0.051 (3)	0.076 (4)	0.052 (3)	−0.022 (3)	−0.001 (3)	0.015 (3)
O13	0.024 (2)	0.038 (3)	0.047 (3)	−0.0011 (19)	0.006 (2)	0.002 (2)
O14	0.040 (3)	0.046 (3)	0.044 (3)	−0.004 (2)	0.010 (2)	0.015 (2)
O15	0.050 (3)	0.047 (3)	0.039 (3)	−0.002 (2)	0.004 (2)	0.008 (2)
O16	0.052 (3)	0.082 (4)	0.055 (3)	−0.016 (3)	0.002 (3)	0.019 (3)
O17	0.051 (3)	0.023 (2)	0.041 (3)	−0.002 (2)	0.016 (2)	−0.002 (2)
O18	0.045 (3)	0.022 (2)	0.046 (3)	−0.002 (2)	0.014 (2)	−0.003 (2)
C1	0.043 (4)	0.027 (3)	0.028 (3)	0.004 (3)	0.012 (3)	0.008 (3)
C2	0.039 (4)	0.030 (3)	0.036 (4)	0.003 (3)	0.011 (3)	−0.002 (3)
C3	0.057 (5)	0.033 (4)	0.051 (4)	−0.005 (3)	0.004 (4)	0.006 (3)
C4	0.051 (5)	0.054 (5)	0.073 (6)	0.007 (4)	−0.002 (4)	0.018 (4)
C5	0.048 (4)	0.059 (5)	0.083 (6)	0.005 (4)	0.006 (4)	−0.004 (5)
C6	0.044 (5)	0.042 (5)	0.103 (7)	−0.007 (4)	0.004 (5)	−0.006 (5)
C7	0.034 (4)	0.034 (4)	0.072 (5)	0.000 (3)	0.013 (4)	0.004 (4)
C8	0.061 (5)	0.096 (6)	0.119 (7)	0.015 (5)	−0.014 (4)	0.002 (6)
C9	0.095 (7)	0.112 (7)	0.119 (7)	0.004 (6)	0.007 (6)	0.005 (7)
C10	0.088 (7)	0.111 (7)	0.116 (7)	0.007 (6)	0.018 (6)	−0.001 (6)
C11	0.091 (7)	0.107 (7)	0.103 (7)	0.002 (6)	0.011 (6)	−0.003 (6)
C9B	0.093 (7)	0.114 (7)	0.112 (7)	0.014 (6)	0.015 (6)	−0.001 (7)
C10B	0.093 (7)	0.110 (7)	0.119 (7)	−0.004 (6)	0.009 (6)	0.000 (7)
C11B	0.093 (7)	0.111 (7)	0.108 (7)	0.007 (6)	0.009 (6)	−0.007 (7)
C12	0.028 (3)	0.031 (3)	0.040 (4)	0.002 (3)	0.011 (3)	0.003 (3)
C13	0.037 (4)	0.032 (3)	0.043 (4)	−0.002 (3)	0.008 (3)	−0.003 (3)
C14	0.033 (4)	0.036 (4)	0.120 (8)	0.002 (3)	0.006 (5)	−0.004 (5)
C15	0.041 (5)	0.051 (6)	0.198 (13)	−0.008 (4)	−0.013 (7)	−0.028 (7)
C16	0.085 (4)	0.073 (5)	0.126 (6)	0.008 (5)	−0.006 (4)	−0.029 (5)
C17	0.054 (5)	0.054 (5)	0.072 (6)	0.007 (4)	−0.001 (4)	−0.001 (4)
C18	0.042 (4)	0.049 (4)	0.045 (4)	−0.005 (3)	−0.004 (3)	0.006 (4)
C19	0.078 (5)	0.096 (6)	0.141 (6)	0.006 (5)	−0.001 (4)	−0.019 (6)
C20	0.090 (5)	0.112 (7)	0.141 (7)	0.011 (6)	0.005 (5)	−0.011 (6)
C21	0.111 (7)	0.111 (7)	0.130 (7)	−0.002 (6)	0.006 (6)	−0.006 (6)
C22	0.093 (7)	0.105 (7)	0.116 (7)	0.007 (6)	0.007 (6)	−0.008 (6)
C20B	0.104 (7)	0.112 (7)	0.142 (7)	0.008 (6)	0.008 (6)	−0.006 (6)
C21B	0.108 (7)	0.116 (7)	0.128 (7)	−0.005 (6)	0.010 (6)	0.004 (6)
C22B	0.099 (7)	0.113 (7)	0.130 (7)	0.003 (6)	0.011 (6)	−0.017 (6)
C23	0.042 (4)	0.033 (4)	0.036 (4)	0.000 (3)	0.014 (3)	0.001 (3)
C24	0.046 (4)	0.028 (3)	0.031 (3)	−0.001 (3)	0.016 (3)	0.000 (3)
C25	0.063 (5)	0.038 (4)	0.047 (4)	−0.015 (4)	0.023 (4)	−0.002 (3)
C26	0.102 (7)	0.045 (5)	0.044 (5)	−0.019 (5)	0.029 (5)	−0.006 (4)
C27	0.090 (6)	0.040 (4)	0.047 (4)	−0.001 (4)	0.035 (4)	0.002 (4)
C28	0.079 (6)	0.040 (4)	0.051 (4)	−0.012 (4)	0.040 (4)	−0.003 (4)
C29	0.058 (5)	0.038 (4)	0.042 (4)	−0.012 (3)	0.023 (4)	−0.007 (3)
C30	0.156 (7)	0.048 (5)	0.065 (5)	−0.008 (5)	0.061 (4)	−0.007 (4)
C31	0.154 (7)	0.084 (6)	0.095 (6)	0.012 (6)	0.070 (5)	−0.007 (5)
C32	0.143 (7)	0.084 (6)	0.083 (5)	−0.004 (5)	0.067 (5)	0.005 (5)
C33	0.166 (7)	0.106 (7)	0.095 (6)	−0.008 (6)	0.043 (5)	−0.002 (6)
C34	0.036 (4)	0.031 (3)	0.033 (3)	−0.001 (3)	0.015 (3)	0.001 (3)
C35	0.047 (4)	0.033 (3)	0.031 (3)	0.000 (3)	0.017 (3)	0.003 (3)
C36	0.057 (4)	0.034 (4)	0.041 (4)	−0.004 (3)	0.022 (3)	0.003 (3)
C37	0.085 (6)	0.045 (4)	0.051 (5)	−0.015 (4)	0.036 (4)	0.001 (4)
C38	0.103 (7)	0.043 (4)	0.043 (4)	−0.007 (5)	0.038 (5)	−0.003 (4)
C39	0.138 (9)	0.044 (5)	0.050 (5)	−0.031 (5)	0.045 (5)	−0.016 (4)
C40	0.083 (6)	0.041 (4)	0.048 (4)	−0.020 (4)	0.032 (4)	−0.004 (4)
C41	0.173 (7)	0.068 (6)	0.074 (6)	−0.003 (6)	0.067 (5)	−0.010 (5)
C42	0.180 (6)	0.124 (7)	0.111 (7)	−0.018 (6)	0.048 (5)	0.001 (6)
C43	0.171 (6)	0.105 (6)	0.101 (6)	−0.005 (5)	0.086 (5)	0.011 (5)
C44	0.162 (7)	0.106 (6)	0.108 (6)	0.016 (6)	0.081 (6)	−0.003 (6)
C45	0.028 (3)	0.035 (4)	0.034 (3)	−0.003 (3)	0.010 (3)	0.000 (3)
C46	0.034 (4)	0.036 (4)	0.040 (4)	−0.004 (3)	0.015 (3)	0.001 (3)
C47	0.041 (4)	0.052 (5)	0.068 (5)	−0.004 (4)	0.008 (4)	0.018 (4)
C48	0.039 (4)	0.058 (5)	0.087 (6)	−0.019 (4)	0.009 (4)	0.002 (5)
C49	0.039 (4)	0.039 (4)	0.113 (7)	−0.010 (4)	0.031 (5)	0.001 (5)
C50	0.058 (5)	0.038 (4)	0.126 (8)	−0.002 (4)	0.030 (5)	0.039 (5)
C51	0.032 (4)	0.055 (5)	0.081 (6)	−0.002 (3)	0.009 (4)	0.032 (4)
C52	0.068 (5)	0.054 (5)	0.146 (8)	−0.021 (4)	0.047 (6)	0.002 (6)
C53	0.093 (6)	0.100 (6)	0.176 (7)	−0.031 (5)	0.040 (6)	0.014 (6)
C54	0.097 (6)	0.061 (5)	0.118 (6)	−0.017 (5)	0.042 (5)	−0.002 (5)
C55	0.129 (7)	0.094 (6)	0.142 (7)	−0.034 (5)	0.069 (6)	0.004 (6)
C56	0.045 (4)	0.035 (4)	0.048 (4)	0.000 (3)	0.014 (4)	0.007 (3)
C57	0.041 (4)	0.037 (4)	0.041 (4)	0.000 (3)	−0.001 (3)	0.005 (3)
C58	0.067 (6)	0.056 (5)	0.055 (5)	−0.016 (4)	0.009 (4)	0.004 (4)
C59	0.090 (7)	0.067 (6)	0.053 (5)	−0.014 (5)	0.016 (5)	0.008 (5)
C60	0.074 (6)	0.054 (5)	0.046 (5)	0.022 (4)	0.003 (4)	0.008 (4)
C61	0.057 (5)	0.092 (7)	0.045 (5)	0.002 (5)	−0.004 (4)	0.004 (5)
C62	0.052 (5)	0.071 (6)	0.054 (5)	−0.012 (4)	0.010 (4)	0.009 (4)
C63	0.141 (6)	0.115 (5)	0.058 (5)	0.024 (6)	0.019 (4)	0.010 (5)
C64	0.171 (6)	0.117 (5)	0.097 (5)	0.030 (5)	0.016 (5)	0.013 (5)
C65	0.144 (6)	0.139 (6)	0.085 (5)	0.022 (5)	0.036 (4)	0.012 (5)
C66	0.152 (6)	0.135 (6)	0.089 (5)	0.009 (5)	0.017 (5)	0.014 (5)
C67	0.044 (4)	0.029 (3)	0.027 (3)	−0.001 (3)	0.015 (3)	0.001 (3)
C68	0.035 (4)	0.035 (4)	0.039 (4)	−0.006 (3)	0.015 (3)	0.000 (3)
C69	0.039 (4)	0.040 (4)	0.090 (6)	−0.007 (3)	0.016 (4)	0.014 (4)
C70	0.047 (5)	0.047 (5)	0.137 (9)	0.001 (4)	0.027 (5)	0.036 (6)
C71	0.055 (5)	0.036 (4)	0.129 (8)	−0.009 (4)	0.040 (6)	0.007 (5)
C72	0.038 (4)	0.066 (6)	0.090 (7)	−0.024 (4)	0.014 (4)	−0.002 (5)
C73	0.041 (4)	0.055 (5)	0.062 (5)	−0.007 (4)	0.012 (4)	0.008 (4)
C74	0.087 (5)	0.062 (6)	0.189 (9)	−0.022 (5)	0.059 (6)	0.015 (7)
C75	0.109 (6)	0.073 (6)	0.150 (7)	−0.024 (5)	0.052 (6)	0.005 (6)
C76	0.136 (7)	0.114 (7)	0.177 (8)	−0.034 (6)	0.071 (6)	0.011 (6)
C77	0.097 (5)	0.110 (7)	0.199 (8)	−0.026 (6)	0.032 (6)	0.015 (6)
C78	0.043 (4)	0.043 (4)	0.051 (4)	−0.001 (3)	0.011 (4)	0.003 (4)
C79	0.047 (4)	0.049 (4)	0.043 (4)	0.004 (4)	0.004 (3)	0.009 (3)
C80	0.054 (5)	0.100 (8)	0.055 (5)	−0.015 (5)	0.002 (4)	0.014 (5)
C81	0.062 (6)	0.117 (9)	0.058 (6)	−0.002 (6)	−0.005 (5)	0.007 (6)
C82	0.096 (8)	0.074 (6)	0.043 (5)	0.024 (6)	0.009 (5)	0.004 (5)
C83	0.096 (8)	0.080 (7)	0.054 (6)	−0.008 (6)	0.021 (5)	0.013 (5)
C84	0.064 (5)	0.066 (6)	0.053 (5)	−0.013 (5)	0.010 (4)	0.004 (4)
C85	0.119 (7)	0.107 (7)	0.063 (5)	0.019 (6)	0.020 (5)	0.008 (5)
C86	0.113 (7)	0.108 (7)	0.079 (7)	0.004 (6)	0.029 (6)	−0.001 (6)
C87	0.128 (7)	0.116 (7)	0.096 (7)	0.007 (7)	0.024 (6)	0.005 (6)
C88	0.116 (7)	0.120 (8)	0.090 (7)	0.010 (7)	0.022 (6)	−0.002 (6)
C86B	0.122 (7)	0.125 (8)	0.095 (7)	0.002 (7)	0.027 (6)	0.008 (7)
C87B	0.113 (7)	0.112 (7)	0.086 (7)	0.008 (6)	0.023 (6)	0.011 (6)
C88B	0.131 (8)	0.121 (8)	0.099 (7)	0.006 (7)	0.022 (6)	−0.003 (7)

### Geometric parameters (Å, °)

**Table:**

Nd1—O7	2.376 (4)	C32—H32C	0.9600
Nd1—O5	2.397 (4)	C33—H33A	0.9600
Nd1—O1	2.405 (4)	C33—H33B	0.9600
Nd1—O3	2.445 (4)	C33—H33C	0.9600
Nd1—O17	2.518 (4)	C34—C35	1.508 (8)
Nd1—O9	2.524 (4)	C35—C36	1.369 (9)
Nd1—O11	2.539 (4)	C35—C40	1.379 (9)
Nd1—O10	2.578 (4)	C36—C37	1.380 (9)
Nd1—O4	2.804 (4)	C36—H36A	0.9300
Nd1—C45	2.926 (6)	C37—C38	1.373 (10)
Nd1—C12	3.013 (6)	C37—H37A	0.9300
Nd2—O6	2.356 (4)	C38—C39	1.402 (11)
Nd2—O8	2.357 (4)	C38—C41	1.527 (11)
Nd2—O4	2.426 (4)	C39—C40	1.360 (10)
Nd2—O2	2.471 (4)	C39—H39A	0.9300
Nd2—O14	2.530 (4)	C40—H40A	0.9300
Nd2—O18	2.536 (4)	C41—C43	1.467 (10)
Nd2—O15	2.544 (5)	C41—C44	1.486 (11)
Nd2—O13	2.572 (4)	C41—C42	1.590 (11)
Nd2—O1	2.761 (4)	C42—H42A	0.9600
Nd2—C67	2.933 (6)	C42—H42B	0.9600
Nd2—C1	3.013 (6)	C42—H42C	0.9600
O1—C1	1.266 (7)	C43—H43A	0.9600
O2—C1	1.262 (7)	C43—H43B	0.9600
O3—C12	1.257 (7)	C43—H43C	0.9600
O4—C12	1.266 (7)	C44—H44A	0.9600
O5—C23	1.259 (8)	C44—H44B	0.9600
O6—C23	1.264 (7)	C44—H44C	0.9600
O7—C34	1.258 (7)	C45—C46	1.488 (8)
O8—C34	1.267 (7)	C46—C47	1.368 (9)
O9—C45	1.260 (7)	C46—C51	1.383 (9)
O10—C45	1.259 (7)	C47—C48	1.394 (10)
O11—C56	1.217 (8)	C47—H47A	0.9300
O12—C56	1.318 (9)	C48—C49	1.363 (12)
O12—H12	0.82 (2)	C48—H48A	0.9300
O13—C67	1.229 (7)	C49—C50	1.359 (11)
O14—C67	1.283 (7)	C49—C52	1.540 (11)
O15—C78	1.213 (8)	C50—C51	1.366 (10)
O16—C78	1.312 (9)	C50—H50A	0.9300
O16—H16	0.82 (2)	C51—H51A	0.9300
O17—H17A	0.8501	C52—C53	1.511 (11)
O17—H17	0.8500	C52—C54	1.515 (10)
O18—H18A	0.8498	C52—C55	1.517 (11)
O18—H18B	0.8501	C53—H53A	0.9600
C1—C2	1.469 (9)	C53—H53B	0.9600
C2—C3	1.380 (9)	C53—H53C	0.9600
C2—C7	1.381 (9)	C54—H54A	0.9600
C3—C4	1.364 (10)	C54—H54B	0.9600
C3—H3A	0.9300	C54—H54C	0.9600
C4—C5	1.395 (11)	C55—H55A	0.9600
C4—H4A	0.9300	C55—H55B	0.9600
C5—C6	1.388 (12)	C55—H55C	0.9600
C5—C8	1.539 (12)	C56—C57	1.466 (9)
C6—C7	1.363 (10)	C57—C62	1.362 (10)
C6—H6A	0.9300	C57—C58	1.383 (10)
C7—H7A	0.9300	C58—C59	1.373 (11)
C8—C9B	1.500 (13)	C58—H58A	0.9300
C8—C9	1.501 (13)	C59—C60	1.370 (12)
C8—C10B	1.505 (13)	C59—H59A	0.9300
C8—C11	1.527 (13)	C60—C61	1.371 (12)
C8—C11B	1.544 (13)	C60—C63	1.516 (12)
C8—C10	1.549 (13)	C61—C62	1.377 (11)
C9—H9A	0.9600	C61—H61A	0.9300
C9—H9B	0.9600	C62—H62A	0.9300
C9—H9C	0.9600	C63—C65	1.498 (11)
C10—H10A	0.9600	C63—C64	1.507 (11)
C10—H10B	0.9600	C63—C66	1.526 (11)
C10—H10C	0.9600	C64—H64A	0.9600
C11—H11A	0.9600	C64—H64B	0.9600
C11—H11B	0.9600	C64—H64C	0.9600
C11—H11C	0.9600	C65—H65A	0.9600
C9B—H9BA	0.9600	C65—H65B	0.9600
C9B—H9BB	0.9600	C65—H65C	0.9600
C9B—H9BC	0.9600	C66—H66A	0.9600
C10B—H10D	0.9600	C66—H66B	0.9600
C10B—H10E	0.9600	C66—H66C	0.9600
C10B—H10F	0.9600	C67—C68	1.480 (8)
C11B—H11D	0.9600	C68—C73	1.371 (9)
C11B—H11E	0.9600	C68—C69	1.387 (10)
C11B—H11F	0.9600	C69—C70	1.377 (10)
C12—C13	1.478 (9)	C69—H69A	0.9300
C13—C18	1.376 (10)	C70—C71	1.369 (12)
C13—C14	1.384 (10)	C70—H70A	0.9300
C14—C15	1.359 (12)	C71—C72	1.389 (12)
C14—H14A	0.9300	C71—C74	1.537 (12)
C15—C16	1.396 (15)	C72—C73	1.386 (10)
C15—H15A	0.9300	C72—H72A	0.9300
C16—C17	1.384 (14)	C73—H73A	0.9300
C16—C19	1.549 (11)	C74—C77	1.490 (11)
C17—C18	1.362 (10)	C74—C76	1.524 (11)
C17—H17B	0.9300	C74—C75	1.529 (11)
C18—H18C	0.9300	C75—H75A	0.9600
C19—C20B	1.507 (14)	C75—H75B	0.9600
C19—C21B	1.509 (14)	C75—H75C	0.9600
C19—C20	1.517 (14)	C76—H76A	0.9600
C19—C21	1.531 (14)	C76—H76B	0.9600
C19—C22	1.534 (13)	C76—H76C	0.9600
C19—C22B	1.539 (14)	C77—H77A	0.9600
C20—H20A	0.9600	C77—H77B	0.9600
C20—H20B	0.9600	C77—H77C	0.9600
C20—H20C	0.9600	C78—C79	1.471 (10)
C21—H21A	0.9600	C79—C80	1.360 (11)
C21—H21B	0.9600	C79—C84	1.373 (11)
C21—H21C	0.9600	C80—C81	1.386 (12)
C22—H22A	0.9600	C80—H80A	0.9300
C22—H22B	0.9600	C81—C82	1.378 (14)
C22—H22C	0.9600	C81—H81A	0.9300
C20B—H20D	0.9600	C82—C83	1.368 (13)
C20B—H20E	0.9600	C82—C85	1.558 (13)
C20B—H20F	0.9600	C83—C84	1.383 (11)
C21B—H21D	0.9600	C83—H83A	0.9300
C21B—H21E	0.9600	C84—H84A	0.9300
C21B—H21F	0.9600	C85—C86B	1.508 (13)
C22B—H22D	0.9600	C85—C88B	1.513 (14)
C22B—H22E	0.9600	C85—C87	1.518 (14)
C22B—H22F	0.9600	C85—C88	1.528 (14)
C23—C24	1.483 (8)	C85—C86	1.540 (13)
C24—C29	1.372 (9)	C85—C87B	1.542 (13)
C24—C25	1.390 (9)	C86—H86D	0.9600
C25—C26	1.363 (10)	C86—H86E	0.9600
C25—H25A	0.9300	C86—H86F	0.9600
C26—C27	1.400 (11)	C87—H87D	0.9600
C26—H26A	0.9300	C87—H87E	0.9600
C27—C28	1.377 (10)	C87—H87F	0.9600
C27—C30	1.504 (10)	C88—H88D	0.9600
C28—C29	1.380 (9)	C88—H88E	0.9600
C28—H28A	0.9300	C88—H88F	0.9600
C29—H29A	0.9300	C86B—H86A	0.9600
C30—C31	1.490 (10)	C86B—H86B	0.9600
C30—C32	1.491 (10)	C86B—H86C	0.9600
C30—C33	1.613 (16)	C87B—H87A	0.9600
C31—H31A	0.9600	C87B—H87B	0.9600
C31—H31B	0.9600	C87B—H87C	0.9600
C31—H31C	0.9600	C88B—H88A	0.9600
C32—H32A	0.9600	C88B—H88B	0.9600
C32—H32B	0.9600	C88B—H88C	0.9600
			
O7—Nd1—O5	134.22 (15)	C27—C28—C29	121.4 (7)
O7—Nd1—O1	76.77 (15)	C27—C28—H28A	119.3
O5—Nd1—O1	73.85 (15)	C29—C28—H28A	119.3
O7—Nd1—O3	81.74 (16)	C24—C29—C28	120.9 (7)
O5—Nd1—O3	87.83 (16)	C24—C29—H29A	119.5
O1—Nd1—O3	125.82 (14)	C28—C29—H29A	119.5
O7—Nd1—O17	74.12 (14)	C31—C30—C32	115.7 (9)
O5—Nd1—O17	144.75 (14)	C31—C30—C27	112.3 (8)
O1—Nd1—O17	140.94 (14)	C32—C30—C27	114.6 (8)
O3—Nd1—O17	74.77 (14)	C31—C30—C33	102.2 (9)
O7—Nd1—O9	137.58 (15)	C32—C30—C33	101.7 (9)
O5—Nd1—O9	84.51 (15)	C27—C30—C33	108.7 (9)
O1—Nd1—O9	105.76 (15)	C30—C31—H31A	109.5
O3—Nd1—O9	123.01 (14)	C30—C31—H31B	109.5
O17—Nd1—O9	80.02 (15)	H31A—C31—H31B	109.5
O7—Nd1—O11	71.42 (16)	C30—C31—H31C	109.5
O5—Nd1—O11	130.64 (16)	H31A—C31—H31C	109.5
O1—Nd1—O11	74.68 (15)	H31B—C31—H31C	109.5
O3—Nd1—O11	141.53 (16)	C30—C32—H32A	109.5
O17—Nd1—O11	71.73 (15)	C30—C32—H32B	109.5
O9—Nd1—O11	68.79 (15)	H32A—C32—H32B	109.5
O7—Nd1—O10	140.41 (14)	C30—C32—H32C	109.5
O5—Nd1—O10	75.34 (14)	H32A—C32—H32C	109.5
O1—Nd1—O10	142.82 (14)	H32B—C32—H32C	109.5
O3—Nd1—O10	72.72 (14)	C30—C33—H33A	109.5
O17—Nd1—O10	70.32 (14)	C30—C33—H33B	109.5
O9—Nd1—O10	50.70 (14)	H33A—C33—H33B	109.5
O11—Nd1—O10	112.00 (14)	C30—C33—H33C	109.5
O7—Nd1—O4	67.62 (14)	H33A—C33—H33C	109.5
O5—Nd1—O4	72.13 (14)	H33B—C33—H33C	109.5
O1—Nd1—O4	77.18 (13)	O7—C34—O8	125.6 (6)
O3—Nd1—O4	48.65 (13)	O7—C34—C35	117.4 (6)
O17—Nd1—O4	113.92 (13)	O8—C34—C35	117.0 (5)
O9—Nd1—O4	154.80 (14)	C36—C35—C40	118.9 (6)
O11—Nd1—O4	134.36 (14)	C36—C35—C34	121.2 (6)
O10—Nd1—O4	112.21 (13)	C40—C35—C34	119.9 (6)
O7—Nd1—C45	144.73 (16)	C35—C36—C37	120.1 (7)
O5—Nd1—C45	80.71 (16)	C35—C36—H36A	120.0
O1—Nd1—C45	127.41 (16)	C37—C36—H36A	120.0
O3—Nd1—C45	97.68 (16)	C38—C37—C36	121.9 (7)
O17—Nd1—C45	71.79 (16)	C38—C37—H37A	119.1
O9—Nd1—C45	25.39 (15)	C36—C37—H37A	119.1
O11—Nd1—C45	89.42 (16)	C37—C38—C39	117.1 (7)
O10—Nd1—C45	25.44 (15)	C37—C38—C41	123.7 (7)
O4—Nd1—C45	136.13 (15)	C39—C38—C41	119.1 (7)
O7—Nd1—C12	73.62 (16)	C40—C39—C38	120.9 (7)
O5—Nd1—C12	79.06 (17)	C40—C39—H39A	119.6
O1—Nd1—C12	101.98 (16)	C38—C39—H39A	119.6
O3—Nd1—C12	23.85 (15)	C39—C40—C35	121.1 (7)
O17—Nd1—C12	94.23 (16)	C39—C40—H40A	119.4
O9—Nd1—C12	142.21 (15)	C35—C40—H40A	119.4
O11—Nd1—C12	144.70 (16)	C43—C41—C44	118.3 (10)
O10—Nd1—C12	92.04 (15)	C43—C41—C38	114.2 (8)
O4—Nd1—C12	24.80 (14)	C44—C41—C38	112.0 (8)
C45—Nd1—C12	117.45 (17)	C43—C41—C42	100.5 (10)
O6—Nd2—O8	133.42 (16)	C44—C41—C42	102.2 (10)
O6—Nd2—O4	77.19 (15)	C38—C41—C42	107.6 (9)
O8—Nd2—O4	73.94 (16)	C41—C42—H42A	109.5
O6—Nd2—O2	81.57 (15)	C41—C42—H42B	109.5
O8—Nd2—O2	87.20 (17)	H42A—C42—H42B	109.5
O4—Nd2—O2	126.61 (14)	C41—C42—H42C	109.5
O6—Nd2—O14	139.94 (15)	H42A—C42—H42C	109.5
O8—Nd2—O14	82.91 (16)	H42B—C42—H42C	109.5
O4—Nd2—O14	104.11 (15)	C41—C43—H43A	109.5
O2—Nd2—O14	122.85 (14)	C41—C43—H43B	109.5
O6—Nd2—O18	74.00 (15)	H43A—C43—H43B	109.5
O8—Nd2—O18	145.04 (15)	C41—C43—H43C	109.5
O4—Nd2—O18	140.59 (14)	H43A—C43—H43C	109.5
O2—Nd2—O18	74.79 (14)	H43B—C43—H43C	109.5
O14—Nd2—O18	82.26 (15)	C41—C44—H44A	109.5
O6—Nd2—O15	72.96 (16)	C41—C44—H44B	109.5
O8—Nd2—O15	130.75 (17)	H44A—C44—H44B	109.5
O4—Nd2—O15	74.84 (15)	C41—C44—H44C	109.5
O2—Nd2—O15	142.01 (16)	H44A—C44—H44C	109.5
O14—Nd2—O15	69.04 (15)	H44B—C44—H44C	109.5
O18—Nd2—O15	71.40 (15)	O10—C45—O9	120.4 (6)
O6—Nd2—O13	140.42 (15)	O10—C45—C46	119.1 (6)
O8—Nd2—O13	75.52 (15)	O9—C45—C46	120.5 (6)
O4—Nd2—O13	142.39 (14)	O10—C45—Nd1	61.7 (3)
O2—Nd2—O13	72.62 (14)	O9—C45—Nd1	59.2 (3)
O14—Nd2—O13	50.38 (14)	C46—C45—Nd1	171.6 (4)
O18—Nd2—O13	70.62 (14)	C47—C46—C51	117.1 (6)
O15—Nd2—O13	110.81 (15)	C47—C46—C45	121.1 (6)
O6—Nd2—O1	67.00 (14)	C51—C46—C45	121.7 (6)
O8—Nd2—O1	71.62 (15)	C46—C47—C48	120.8 (7)
O4—Nd2—O1	77.67 (14)	C46—C47—H47A	119.6
O2—Nd2—O1	48.94 (13)	C48—C47—H47A	119.6
O14—Nd2—O1	153.03 (14)	C49—C48—C47	121.8 (8)
O18—Nd2—O1	113.82 (13)	C49—C48—H48A	119.1
O15—Nd2—O1	135.32 (14)	C47—C48—H48A	119.1
O13—Nd2—O1	112.52 (13)	C50—C49—C48	116.5 (7)
O6—Nd2—C67	145.94 (16)	C50—C49—C52	119.6 (8)
O8—Nd2—C67	80.13 (16)	C48—C49—C52	123.9 (8)
O4—Nd2—C67	126.68 (16)	C49—C50—C51	123.1 (8)
O2—Nd2—C67	97.01 (16)	C49—C50—H50A	118.5
O14—Nd2—C67	25.84 (16)	C51—C50—H50A	118.5
O18—Nd2—C67	72.84 (15)	C50—C51—C46	120.7 (7)
O15—Nd2—C67	89.22 (17)	C50—C51—H51A	119.7
O13—Nd2—C67	24.71 (15)	C46—C51—H51A	119.7
O1—Nd2—C67	135.40 (15)	C53—C52—C54	109.3 (9)
O6—Nd2—C1	72.95 (16)	C53—C52—C55	108.4 (9)
O8—Nd2—C1	78.58 (17)	C54—C52—C55	108.0 (9)
O4—Nd2—C1	102.51 (16)	C53—C52—C49	110.5 (8)
O2—Nd2—C1	24.10 (15)	C54—C52—C49	109.4 (7)
O14—Nd2—C1	141.71 (15)	C55—C52—C49	111.1 (8)
O18—Nd2—C1	94.16 (15)	C52—C53—H53A	109.5
O15—Nd2—C1	145.50 (16)	C52—C53—H53B	109.5
O13—Nd2—C1	92.35 (14)	H53A—C53—H53B	109.5
O1—Nd2—C1	24.83 (14)	C52—C53—H53C	109.5
C67—Nd2—C1	116.98 (17)	H53A—C53—H53C	109.5
C1—O1—Nd1	167.8 (4)	H53B—C53—H53C	109.5
C1—O1—Nd2	88.8 (4)	C52—C54—H54A	109.5
Nd1—O1—Nd2	103.47 (14)	C52—C54—H54B	109.5
C1—O2—Nd2	102.8 (4)	H54A—C54—H54B	109.5
C12—O3—Nd1	104.3 (4)	C52—C54—H54C	109.5
C12—O4—Nd2	171.4 (4)	H54A—C54—H54C	109.5
C12—O4—Nd1	86.9 (4)	H54B—C54—H54C	109.5
Nd2—O4—Nd1	101.68 (15)	C52—C55—H55A	109.5
C23—O5—Nd1	137.8 (4)	C52—C55—H55B	109.5
C23—O6—Nd2	141.2 (4)	H55A—C55—H55B	109.5
C34—O7—Nd1	138.8 (4)	C52—C55—H55C	109.5
C34—O8—Nd2	138.9 (4)	H55A—C55—H55C	109.5
C45—O9—Nd1	95.4 (4)	H55B—C55—H55C	109.5
C45—O10—Nd1	92.9 (4)	O11—C56—O12	122.3 (7)
C56—O11—Nd1	144.6 (5)	O11—C56—C57	122.5 (6)
C56—O12—H12	113 (7)	O12—C56—C57	115.1 (6)
C67—O13—Nd2	94.3 (4)	C62—C57—C58	118.0 (7)
C67—O14—Nd2	94.9 (4)	C62—C57—C56	123.3 (7)
C78—O15—Nd2	144.6 (5)	C58—C57—C56	118.6 (7)
C78—O16—H16	111 (8)	C59—C58—C57	120.2 (8)
Nd1—O17—H17A	109.4	C59—C58—H58A	119.9
Nd1—O17—H17	135.0	C57—C58—H58A	119.9
H17A—O17—H17	96.0	C60—C59—C58	122.2 (9)
Nd2—O18—H18A	114.5	C60—C59—H59A	118.9
Nd2—O18—H18B	103.9	C58—C59—H59A	118.9
H18A—O18—H18B	107.1	C59—C60—C61	116.8 (8)
O2—C1—O1	119.5 (6)	C59—C60—C63	120.5 (9)
O2—C1—C2	120.2 (5)	C61—C60—C63	122.7 (9)
O1—C1—C2	120.3 (6)	C60—C61—C62	121.8 (8)
O2—C1—Nd2	53.1 (3)	C60—C61—H61A	119.1
O1—C1—Nd2	66.4 (3)	C62—C61—H61A	119.1
C2—C1—Nd2	173.2 (4)	C57—C62—C61	120.9 (8)
C3—C2—C7	117.4 (6)	C57—C62—H62A	119.5
C3—C2—C1	120.8 (6)	C61—C62—H62A	119.5
C7—C2—C1	121.7 (6)	C65—C63—C64	112.0 (11)
C4—C3—C2	121.5 (7)	C65—C63—C60	111.4 (9)
C4—C3—H3A	119.2	C64—C63—C60	110.6 (9)
C2—C3—H3A	119.2	C65—C63—C66	105.1 (10)
C3—C4—C5	121.1 (8)	C64—C63—C66	105.0 (10)
C3—C4—H4A	119.4	C60—C63—C66	112.4 (10)
C5—C4—H4A	119.4	C63—C64—H64A	109.5
C6—C5—C4	116.9 (8)	C63—C64—H64B	109.5
C6—C5—C8	122.4 (8)	H64A—C64—H64B	109.5
C4—C5—C8	120.7 (8)	C63—C64—H64C	109.5
C7—C6—C5	121.4 (8)	H64A—C64—H64C	109.5
C7—C6—H6A	119.3	H64B—C64—H64C	109.5
C5—C6—H6A	119.3	C63—C65—H65A	109.5
C6—C7—C2	121.4 (7)	C63—C65—H65B	109.5
C6—C7—H7A	119.3	H65A—C65—H65B	109.5
C2—C7—H7A	119.3	C63—C65—H65C	109.5
C9B—C8—C10B	115.4 (17)	H65A—C65—H65C	109.5
C9—C8—C11	113.2 (16)	H65B—C65—H65C	109.5
C9B—C8—C5	108.8 (12)	C63—C66—H66A	109.5
C9—C8—C5	114.0 (12)	C63—C66—H66B	109.5
C10B—C8—C5	111.9 (12)	H66A—C66—H66B	109.5
C11—C8—C5	109.6 (11)	C63—C66—H66C	109.5
C9B—C8—C11B	102.9 (15)	H66A—C66—H66C	109.5
C10B—C8—C11B	107.8 (16)	H66B—C66—H66C	109.5
C5—C8—C11B	109.5 (12)	O13—C67—O14	119.6 (6)
C9—C8—C10	103.1 (16)	O13—C67—C68	121.2 (6)
C11—C8—C10	109.8 (15)	O14—C67—C68	119.0 (6)
C5—C8—C10	106.7 (12)	O13—C67—Nd2	61.0 (3)
C8—C9—H9A	109.5	O14—C67—Nd2	59.3 (3)
C8—C9—H9B	109.5	C68—C67—Nd2	168.4 (4)
C8—C9—H9C	109.5	C73—C68—C69	117.2 (7)
C8—C10—H10A	109.5	C73—C68—C67	122.9 (6)
C8—C10—H10B	109.5	C69—C68—C67	119.9 (6)
C8—C10—H10C	109.5	C70—C69—C68	120.9 (7)
C8—C11—H11A	109.5	C70—C69—H69A	119.5
C8—C11—H11B	109.5	C68—C69—H69A	119.5
C8—C11—H11C	109.5	C71—C70—C69	122.0 (8)
C8—C9B—H9BA	109.5	C71—C70—H70A	119.0
C8—C9B—H9BB	109.5	C69—C70—H70A	119.0
H9BA—C9B—H9BB	109.5	C70—C71—C72	117.5 (7)
C8—C9B—H9BC	109.5	C70—C71—C74	120.4 (9)
H9BA—C9B—H9BC	109.5	C72—C71—C74	122.2 (8)
H9BB—C9B—H9BC	109.5	C73—C72—C71	120.4 (8)
C8—C10B—H10D	109.5	C73—C72—H72A	119.8
C8—C10B—H10E	109.5	C71—C72—H72A	119.8
H10D—C10B—H10E	109.5	C68—C73—C72	122.0 (8)
C8—C10B—H10F	109.5	C68—C73—H73A	119.0
H10D—C10B—H10F	109.5	C72—C73—H73A	119.0
H10E—C10B—H10F	109.5	C77—C74—C76	105.3 (10)
C8—C11B—H11D	109.5	C77—C74—C75	113.2 (11)
C8—C11B—H11E	109.5	C76—C74—C75	106.9 (10)
H11D—C11B—H11E	109.5	C77—C74—C71	113.1 (9)
C8—C11B—H11F	109.5	C76—C74—C71	109.3 (10)
H11D—C11B—H11F	109.5	C75—C74—C71	108.8 (8)
H11E—C11B—H11F	109.5	C74—C75—H75A	109.5
O3—C12—O4	120.1 (6)	C74—C75—H75B	109.5
O3—C12—C13	119.3 (6)	H75A—C75—H75B	109.5
O4—C12—C13	120.5 (6)	C74—C75—H75C	109.5
O3—C12—Nd1	51.8 (3)	H75A—C75—H75C	109.5
O4—C12—Nd1	68.3 (3)	H75B—C75—H75C	109.5
C13—C12—Nd1	170.9 (4)	C74—C76—H76A	109.5
C18—C13—C14	118.2 (7)	C74—C76—H76B	109.5
C18—C13—C12	120.8 (6)	H76A—C76—H76B	109.5
C14—C13—C12	120.9 (6)	C74—C76—H76C	109.5
C15—C14—C13	119.5 (8)	H76A—C76—H76C	109.5
C15—C14—H14A	120.2	H76B—C76—H76C	109.5
C13—C14—H14A	120.2	C74—C77—H77A	109.5
C14—C15—C16	123.5 (9)	C74—C77—H77B	109.5
C14—C15—H15A	118.3	H77A—C77—H77B	109.5
C16—C15—H15A	118.3	C74—C77—H77C	109.5
C17—C16—C15	115.2 (9)	H77A—C77—H77C	109.5
C17—C16—C19	121.5 (11)	H77B—C77—H77C	109.5
C15—C16—C19	123.2 (10)	O15—C78—O16	122.2 (7)
C18—C17—C16	122.2 (9)	O15—C78—C79	123.1 (7)
C18—C17—H17B	118.9	O16—C78—C79	114.7 (7)
C16—C17—H17B	118.9	C80—C79—C84	119.7 (8)
C17—C18—C13	121.2 (7)	C80—C79—C78	122.1 (7)
C17—C18—H18C	119.4	C84—C79—C78	118.2 (7)
C13—C18—H18C	119.4	C79—C80—C81	120.1 (9)
C20B—C19—C21B	118.5 (18)	C79—C80—H80A	120.0
C20—C19—C21	113.5 (18)	C81—C80—H80A	120.0
C20—C19—C22	114.8 (16)	C82—C81—C80	121.1 (9)
C21—C19—C22	103.6 (17)	C82—C81—H81A	119.4
C20B—C19—C22B	101.1 (17)	C80—C81—H81A	119.4
C21B—C19—C22B	109.0 (17)	C83—C82—C81	117.8 (8)
C20B—C19—C16	108.6 (13)	C83—C82—C85	121.2 (10)
C21B—C19—C16	109.6 (14)	C81—C82—C85	120.9 (10)
C20—C19—C16	104.8 (14)	C82—C83—C84	121.5 (9)
C21—C19—C16	105.7 (14)	C82—C83—H83A	119.2
C22—C19—C16	114.3 (13)	C84—C83—H83A	119.2
C22B—C19—C16	109.5 (14)	C79—C84—C83	119.8 (8)
C19—C20—H20A	109.5	C79—C84—H84A	120.1
C19—C20—H20B	109.5	C83—C84—H84A	120.1
C19—C20—H20C	109.5	C86B—C85—C88B	111.1 (17)
C19—C21—H21A	109.5	C87—C85—C88	110.1 (16)
C19—C21—H21B	109.5	C87—C85—C86	108.4 (16)
H21A—C21—H21B	109.5	C88—C85—C86	105.1 (15)
C19—C21—H21C	109.5	C86B—C85—C87B	109.9 (16)
H21A—C21—H21C	109.5	C88B—C85—C87B	108.3 (16)
H21B—C21—H21C	109.5	C86B—C85—C82	111.5 (13)
C19—C22—H22A	109.5	C88B—C85—C82	108.7 (13)
C19—C22—H22B	109.5	C87—C85—C82	110.4 (12)
C19—C22—H22C	109.5	C88—C85—C82	113.3 (13)
C19—C20B—H20D	109.5	C86—C85—C82	109.3 (12)
C19—C20B—H20E	109.5	C87B—C85—C82	107.2 (11)
H20D—C20B—H20E	109.5	C85—C86—H86D	109.5
C19—C20B—H20F	109.5	C85—C86—H86E	109.5
H20D—C20B—H20F	109.5	H86D—C86—H86E	109.5
H20E—C20B—H20F	109.5	C85—C86—H86F	109.5
C19—C21B—H21D	109.5	H86D—C86—H86F	109.5
C19—C21B—H21E	109.5	H86E—C86—H86F	109.5
H21D—C21B—H21E	109.5	C85—C87—H87D	109.5
C19—C21B—H21F	109.5	C85—C87—H87E	109.5
H21D—C21B—H21F	109.5	C85—C87—H87F	109.5
H21E—C21B—H21F	109.5	C85—C88—H88D	109.5
C19—C22B—H22D	109.5	C85—C88—H88E	109.5
C19—C22B—H22E	109.5	C85—C88—H88F	109.5
H22D—C22B—H22E	109.5	C85—C86B—H86A	109.5
C19—C22B—H22F	109.5	C85—C86B—H86B	109.5
H22D—C22B—H22F	109.5	H86A—C86B—H86B	109.5
H22E—C22B—H22F	109.5	C85—C86B—H86C	109.5
O5—C23—O6	124.2 (6)	H86A—C86B—H86C	109.5
O5—C23—C24	119.0 (6)	H86B—C86B—H86C	109.5
O6—C23—C24	116.8 (6)	C85—C87B—H87A	109.5
C29—C24—C25	118.2 (6)	C85—C87B—H87B	109.5
C29—C24—C23	122.1 (6)	H87A—C87B—H87B	109.5
C25—C24—C23	119.7 (6)	C85—C87B—H87C	109.5
C26—C25—C24	121.1 (7)	H87A—C87B—H87C	109.5
C26—C25—H25A	119.4	H87B—C87B—H87C	109.5
C24—C25—H25A	119.4	C85—C88B—H88A	109.5
C25—C26—C27	121.0 (7)	C85—C88B—H88B	109.5
C25—C26—H26A	119.5	H88A—C88B—H88B	109.5
C27—C26—H26A	119.5	C85—C88B—H88C	109.5
C28—C27—C26	117.5 (7)	H88A—C88B—H88C	109.5
C28—C27—C30	123.2 (7)	H88B—C88B—H88C	109.5
C26—C27—C30	119.4 (7)		

### Hydrogen-bond geometry (Å, °)

**Table:**

*D*—H···*A*	*D*—H	H···*A*	*D*···*A*	*D*—H···*A*
O17—H17A···O13^i^	0.85	2.12	2.870 (6)	148
O17—H17···O2^i^	0.85	1.96	2.786 (6)	165
O18—H18A···O3^ii^	0.85	2.01	2.782 (6)	151
O18—H18B···O10^ii^	0.85	2.14	2.843 (6)	140

Symmetry codes: (i) *x*, *y*−1, *z*; (ii) *x*, *y*+1, *z*.
